# AAV process intensification by perfusion bioreaction and integrated clarification

**DOI:** 10.3389/fbioe.2022.1020174

**Published:** 2022-11-07

**Authors:** João P. Mendes, Bárbara Fernandes, Earl Pineda, Shashi Kudugunti, Mike Bransby, René Gantier, Cristina Peixoto, Paula M. Alves, António Roldão, Ricardo J. S. Silva

**Affiliations:** ^1^ iBET, Instituto de Biologia Experimental e Tecnológica, Oeiras, Portugal; ^2^ Instituto de Tecnologia Química e Biológica António Xavier, Universidade Nova de Lisboa, Oeiras, Portugal; ^3^ Repligen, Waltham, MA, United States

**Keywords:** adeno-associated virus, alternating tangential flow, perfusion, gene therapy, integrated manufacturing, tangential flow depth filtration, process intensification

## Abstract

Adeno-associated viruses (AAVs) demand for clinical trials and approved therapeutic applications is increasing due to this vector’s overall success and potential. The high doses associated with administration strategies challenges bioprocess engineers to develop more efficient technologies and innovative strategies capable of increasing volumetric productivity. In this study, alternating tangential flow (ATF) and Tangential Flow Depth filtration (TFDF) techniques were compared as to their potential for 1) implementing a high-cell-density perfusion process to produce AAV8 using mammalian HEK293 cells and transient transfection, and 2) integrating AAV harvest and clarification units into a single step. On the first topic, the results obtained demonstrate that AAV expression improves with a medium exchange strategy. This was evidenced firstly in the small-scale perfusion-mocking study and later verified in the 2 L bioreactor operated in perfusion mode. Fine-tuning the shear rate in ATF and TFDF proved instrumental in maintaining high cell viabilities and, most importantly, enhancing AAV-specific titers (7.6 × 10^4^ VG/cell), i.e., up to 4-fold compared to non-optimized perfusion cultures and 2-fold compared with batch operation mode. Regarding the second objective, TFDF enabled the highest recovery yields during perfusion-based continuous harvest of extracellular virus and lysate clarification. This study demonstrates that ATF and TFDF techniques have the potential to support the production and continuous harvest of AAV, and enable an integrated clarification procedure, contributing to the simplification of operations and improving manufacturing efficiency.

## 1 Introduction

The approval of gene therapies such as Luxturna (Spark Therapeutics) and Zolgensma (Novartis) has pushed adeno-associated viruses (AAV) to the clinic (Merten et al., 2014; Mendell et al., 2021). However, the quantity of AAV required for such applications varies from 1 × 10^13^ (e.g., Leber’s congenital amaurosis) to 5 × 10^20^ viral genomes (e.g., Duchenne muscular dystrophy), placing substantial pressure on manufacturing processes (Koilkonda et al., 2014; Crudele and Chamberlain, 2019). Despite the several technologies available, the upstream processing of AAV is still one of the main bottlenecks of clinical-grade AAV manufacturing (Smith et al., 2018) with specific production titers and vector quality (i.e., % full particles) being two of the most challenging parameters to control and/or optimize (Merten, 2016). The implementation of process intensification strategies has already been demonstrated to overcome these challenges with successful case studies for monoclonal antibodies (Chen et al., 2018; Xu et al., 2020). These methodologies enable high cell density (HCD) cultures, increased productivity, and the reduction of processing times required to achieve higher target quantities (Chahal et al., 2014; Yang et al., 2014; Cook et al., 2020).

Fed-batch cultures have been extensively demonstrated to improve cell growth and viability while enabling higher yields for protein and virus-like particle production (Chan et al., 2002; Meghrous et al., 2009; Cao et al., 2019). Additionally, perfusion has been successfully implemented for increasing cell densities and productivities. This is, accomplished using devices that enable cell retention and simultaneous medium exchange thus preventing nutrient depletion while removing growth-inhibiting compounds (Cameau et al., 2019). Amongst the different cell retention devices available, ATF (alternating tangential flow) has been widely used for bioprocess intensification (Hadpe et al., 2017; Kamga et al., 2018; Fernandes et al., 2021). The ATF system uses a diaphragm pump to create cycles composed of alternating pressure and exhaust periods. Contrarily to conventional tangential flow filtration, fluid flow direction in ATF is reversed during the exhaust cycle. This promotes a potential backflush of the membrane reducing fouling while maintaining a stable flux for a longer duration.

Recently, a new filter technology has been developed—tangential flow depth filtration (TFDF). It consists of an elongated tubular depth filter, with a 2–5 µm average pore rating, operated in tangential flow mode that enables the benefits of both filtration strategies, i.e., tangential, and depth filtration. Therefore, considering the properties of hollow fibers and related devices, some studies have already been made on their applicability for bulk clarification (Hadpe et al., 2017; Raghavan et al., 2019). The tangential nature and pore size of TFDF filters potentiate their use as cell retention devices in perfusion cell culture, theoretically enabling continuous harvesting of AAV. In addition to this, the same filter device could be used for clarifying cell lysates thus integrating AAV production and clarification in a single unitary operation. A continuous AAV harvest procedure using ATF and TFDF driven by a continuous withdrawal of permeate can be envisioned using these technologies. Such strategies should balance the duration of transient stages of AAV expression, membrane sieving effects, and dilution of outlet material streams as a consequence of the imposed perfusion. Importantly, they can also contribute to reducing equipment and unitary operations footprint, thus positively impacting process economics.

In this work, two different cell retention devices (XCell ATF^®^ and Krosflo TFDF^®^) were evaluated as to their potential to implement a continuous, integrated AAV production process. We started by implementing batch 2 L bioreactors to benchmark both AAV production and clarification with standard strategies. Given the results of specific cell titers, shake-flask experiments were performed, in both batch and perfusion-mocking scenarios, to investigate the impact of medium exchange on cell culture kinetics, the potential of high cell density, and the effects of these changes on virus production. The AAV productions were afterwards scaled to 2 L perfusion cultures in controlled stirred tank bioreactors to assess the performance of ATF and TFDF in promoting high cell densities and viabilities to optimize AAV8 production. Finally, the applicability of integrating AAV8 production with the initial steps of downstream processing—harvest and clarification—was evaluated.

## 2 Experimental methods

### 2.1 Cell culture

Human Embryonic Kidney cells 293T (HEK 293T), adapted to suspension, were purchased from ATCC (ACS-4500). These were routinely sub-cultured to 0.6 × 10^6^ cells/mL every 48–72 h when cell concentration reached 2–3 × 10^6^ cells/mL using vented non-baffled shake flasks with BalanCD HEK293 medium (Irvine Scientific) supplemented with 4 mM of GlutaMAX (Gibco) under a humidified atmosphere of 5% CO_2_ in air at 37°C with controlled agitation (orbital diameter of 25 mm, 90 rpm).

### 2.2 Adeno-associated virus production

#### 2.2.1 Shake flask cultures

Shake flask (SF) cultures were performed aiming to mock batch (set A) and perfusion (set B) bioreactor cultures. For set A, cells were inoculated at 0.6 × 10^6^ cells/mL and cultured until reaching desired concentrations for transfection (2 × 10^6^, 5 × 10^6^ and 10 × 10^6^ cells/mL). For set B, cell cultures from a seed train were centrifuged at ×300 g for 10 min and resuspended in fresh medium at specific concentrations (2 × 10^6^, 5 × 10^6^, and 10 × 10^6^ cells/mL) before transfection.

#### 2.2.2 Stirred-tank bioreactor cultures

Cultures were performed in a 2 L Biostat^®^ D-DCU (Sartorius) stirred-tank bioreactor (STB) equipped with two Rushton impellers and a ring-sparger for gas supply. The pO2 was set to 40% of air saturation and was maintained by varying the agitation rate (70–200 rpm), the percentage of O_2_ in the gas mixture (0%–100%), and gas flow rate (0.01–0.04 vvm). The pH value was maintained by the automatic addition of either 1 M of Na_3_CO or CO_2_ within the gas mix.

For batch cultures, cells were inoculated at 0.6 × 10^6^ cells/mL and transfected (according to Section 2.2.3) when viable cell concentration (VCC) reached the target value (2 × 10^6^ or 5 × 10^6^ cells/mL). Cell culture was carried out until cell viability dropped below 70%, being subject to cell lysis and clarification as described in Section 2.3 and Section 2.4.

For perfusion cultures, STB were coupled to either an XCell ATF (Repligen) or a Krosflo TFDF (Repligen) system. The microfiltration polyethersulfone hollow fiber module for the ATF-2 system had a lumen internal diameter of 1.0 mm and 1300 cm^2^ of surface area; the TFDF device had a pore rating of 2.0–5.0 µm and a surface area of 30 cm^2^. Perfusion cultures were performed using the same cell culture setup as in batch and maintained with a similar perfusion rate of 1 day^−1^, starting 48 h after inoculation. Perfusion was halted during transfection for a period of 4 h, being resumed for an additional 24 h period. After this, the perfusion rate was reduced to 0.5 day^−1^ until the end of the culture, determined by a defined endpoint of 80% of viable cells.

#### 2.2.3 Transfection protocol

Cells were transfected with a DNA plasmid solution containing 1.5 µg of total plasmid DNA per 10^6^ cells. This mix included pHelper:pAAV-RC:pAAV-GFP at a molar ratio of 1:1:1 diluted in a specific volume of supplemented culture medium, corresponding to 5% of culture volume. Additionally, PEI MAX (PolySciences) transfection reagent was added with a 1:2 µg DNA/ug PEI ratio between total plasmid and reagent. This solution was incubated at room temperature for up to 15 min before addition.

### 2.3 Cell lysis

Cells were lysed with 50 mM TRIS, 0.1% Triton X-100 (Sigma Aldrich), and 2 mM of MgCl_2_ followed by the addition of 50 Units per mL of Benzonase (Merck Millipore). To prevent aggregation, salt-concentrated solutions of MgSO_4_ and NaCl were supplemented to a final concentration of 37.5 and 400 mM, respectively.

### 2.4 Harvest and clarification

For batch cultures, the cell lysate was harvested and clarified with two different filter trains. The first consisted of a 5.0 µm ULTA GF filter (Cytiva) followed by a second filtration stage with 0.8/0.2 µm Sartopore 2 XLG (Sartorius Stedim Biotech). For the second filter train, a TFDF device (30 cm^2^) (Repligen) was used before a Millistak X0SP (Merck Millipore). The filters were previously rinsed with mili-Q water and a buffer solution (50 mM TRIS, 400 mM NaCl, pH 8.0) and operated under manufacturer-recommended guidelines.

Perfusion cultures were clarified using a three-step process. First, extracellular AAV were harvested using the cell retention device implemented in the bioreactor. Permeate flow rate was ramped up to 22.5 mL/min and fresh cell culture media was fed to the bioreactor. After exchanging one volume of culture (2 L), the second step—cell lysis (according to Section 2.3) was carried out. During this procedure, the permeate flow rate was halted. The third and final step of the harvest and clarification procedure was carried out by setting the permeate flow rate of ATF and TFDF to 22.5 mL/min. Recirculation rate was defined at 0.5 L/min for ATF whereas for TFDF pump rate was automatedly adjusted to provide a shear rate of 6000 s^−1^, except for TFDF1 in which a shear rate of 4000 s^−1^ was used. The cell lysates were filtered using the cell retention devices implemented in the bioreactor. The clarified bulks using TFDF were subject to a second filtration step using either a Sartopore 2 XLG or a Millistak X0SP, before being stored at −80°C.

### 2.5 Analytical methods

#### 2.5.1 Turbidity measurement

The turbidity of cell lysates and clarified samples were measured using a turbidimeter (2100 Qis Portable, HACH).

#### 2.5.2 Viable cell concentration

Cell concentration and viability were quantified using the Cedex HiRes Analyzer (Roche) and Vi-CELL BLU (Beckman Coulter) using the manufacturer’s instructions.

#### 2.5.3 Virus titer quantification

Total particle (TP) quantification was performed with conformational AAV8 ELISA XPRES kit (PROGEN) according to the manufacturer’s instruction. Samples were diluted in working buffer and applied in triplicate. Absorbance measurements were obtained at 450 nm, using 650 nm as a reference, on Infinite^®^ PRO NanoQuant (Tecan) microplate reader.

Viral genome (VG) copies were quantified by qPCR. DNA was extracted using the High Pure Viral Nucleic Acid Kit (Roche). qPCR was performed with a probe (5′-TTG​CCG​TCC​TCC​TTG​AAG​TCG​AT-3′) and transgene-specific primers (forward primer, 5′-GAA​CCG​CAT​CGA​GCT​GAA-3′ and reverse primer, 5′-TGC​TTG​TCG​GCC​ATG​ATA​TAG-3′). The quantification was performed using the LightCycler system (Roche Diagnostic) using the eGFP transgene plasmid as standard.

## 3 Results

### 3.1 Adeno-associated virus production in batch mode

**FIGURE 1:**
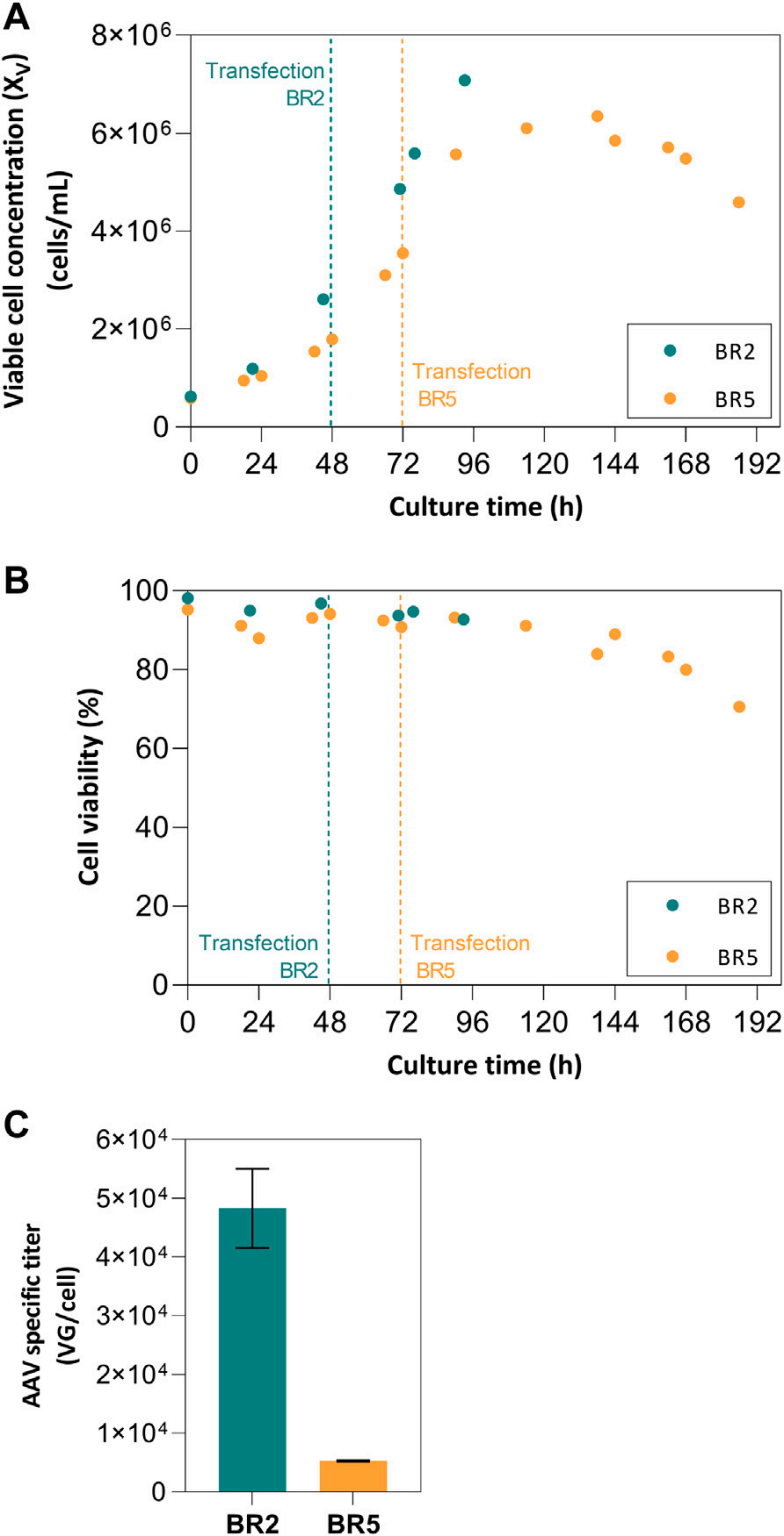
Batch stirred-tank bioreactor (2 L) analysis. **(A)** Cell growth profiles for batch runs with VCC^TOT^ of 2 × 10^6^ cells/mL (BR2) and VCC^TOT^ of 5 × 10^6^ cells/mL (BR5); **(B)** Viability profiles for bioreactor batch runs BR2 and BR5; **(C)** Specific titers for each run.

The impact of cell concentration at transfection (2 × 10^6^ vs. 5 × 10^6^ cells/mL) on AAV production was assessed in 2 L STB operated in batch mode. Cell growth kinetics was similar in both production runs, with exponential cell growth until transfection time, increase in cell concentration upon transfection for additional 48 h, and onset of cell viability drop afterwards (Figure 1A,B). Regarding virus production, the bioreactor in which cells were transfected at 2 × 10^6^ cells/mL (named “BR2” from now on) returned an approximate 1-log higher AAV titer per cell in comparison to the bioreactor in which cells were transfected at 5 × 10^6^ cell/mL (named “BR5” from now on)—Figure 1C. Follow-up studies were performed in SF cultures to 1) confirm the cell density effect observed at bioreactor scale, and 2) evaluate the impact of medium exchange on AAV titers (i.e., perfusion-mocking study).

#### 3.1.1 Impact of medium exchange on adeno-associated virus production

**FIGURE 2:**
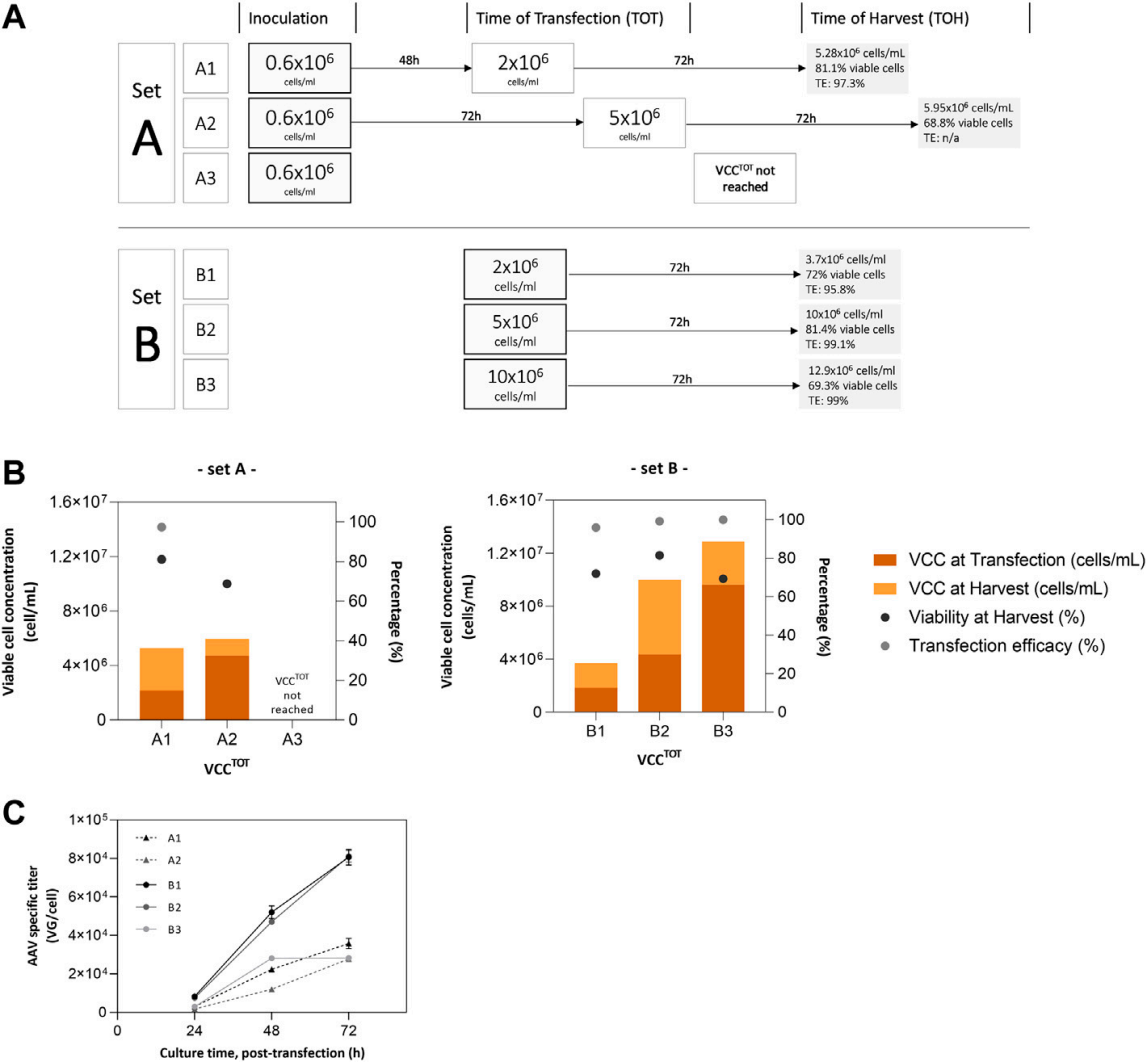
Small-scale experiments for AAV production. **(A)** batch culture condition (set A) and perfusion-mocking condition (set B); **(B)** Cell culture parameters (VCC at transfection and harvest, cell viability and transfection efficacy); **(C)** Cumulative virus yield (intra + extra) for sets A and B; no data available for VCC^ToT^—10 × 10^6^ cells/mL of set A as cells did not reach the defined concentration value for transfection. For set A, cells were inoculated at 0.6 × 10^6^ cells/mL and maintained until reaching the designated viable cell concentration at the time of transfection. For set B, cultures were inoculated at respective VCC^TOT^, after medium exchange and concentration through centrifugation. Transfection was performed at VCC^TOT^ for set A, and 1 h post-inoculation for set B. AAV were harvested at 72 hours post-transfection.

To evaluate the impact of the seed train strategy and cell concentration at the time of transfection (TOT) on AAV production, two sets of SF cultures were prepared. Set A consists of a standard batch process where cells are cultured until reaching desired concentration for transfection; set B mimics a perfusion culture where cells are resuspended in fresh culture medium at a determined concentration and transfected immediately (Figure 2A).

Transfection efficiency was above 95% in all experiments reported (Figure 2B). Cell growth kinetics developed as expected for a transfection-based process, with cell concentration increasing after transfection as it is observable by the higher VCC obtained in harvest (Figure 2B). The exception is condition A3, in which desired cell concentration at transfection could not be reached.

The analysis of the cumulative titers (intracellular plus extracellular fractions) at 72 h of production (Figure 2C) confirms the cell density effect observed at the bioreactor scale, i.e., increasing viable cell concentration at the time of transfection (VCC^TOT^) leads to a reduction in AAV titers per cell. More importantly, it shows that the virus yields obtained in set B (i.e., perfusion-mocking process) are 2–3-fold higher than those for set A (i.e., batch process), except for B3 run that reports titers similar to those achieved in set A (approx. 3 × 10^4^ VG/cell).

Based on these results, subsequent studies will target perfusion cultures at 2 L scale using VCC^TOT^ above 5 × 10^6^ cells/mL to implement with different cell retention devices towards continuous production and integrated harvest of AAV.

### 3.2 Adeno-associated virus production in perfusion mode

The impact of perfusion on AAV titers was assessed using two different cell retention devices (ATF *vs.* TFDF) and two VCC^ToT^ (5 × 10^6^ and 10 × 10^6^ cells/mL). Table 1 lists the different perfusion runs performed and operating conditions.

**TABLE 1:** Parameters used in the ATF and TFDF runs performed.

Bioreactor	VCC^TOT^ (cells/mL)	Shear rate (s^−1^)	Filter area (cm^2^)	Pore size (µm)
ATF1	10×10^6^	2100^a^/670^b^	1300	0.5
ATF2	5×10^6^	670	1300	0.5
ATF3	5×10^6^	670	1300	0.2
TFDF1	10×10^6^	2100	30	5.0
TFDF2	5×10^6^	2100	30	5.0
TFDF3	5×10^6^	500	30	5.0

^a^ : 0–48 h post inoculation.

^b^ : > 48 h post inoculation.

#### 3.2.1 Adeno-associated virus production process using ATF

**FIGURE 3:**
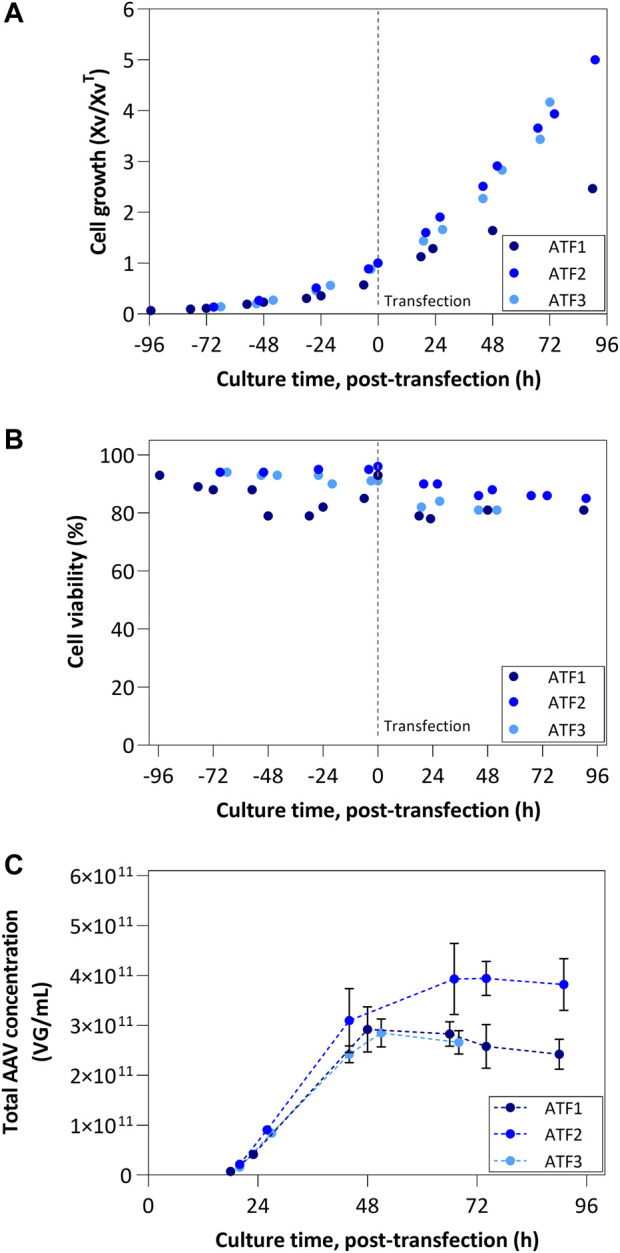
ATF process results: **(A)** Cell growth profiles and **(B)** Viability profiles; ATF1 was transfected at 10 × 10^6^ cells/mL, while remaining runs were transfected at 5 × 10^6^ cells/mL; Cell growth is represented by normalizing viable cell concentration by the VCC at the time of transfection. **(C)** Concentration of total AAV produced considering intracellular, extracellular and permeate fractions.

The normalized cell growth profiles for all ATF runs are similar until TOT; from this point onwards, ATF1 shows a lower cell growth rate when compared to the other ATF runs (Figure 3A). Likewise, notwithstanding a small drop in cell viability at 48 h post-inoculation promoted by the high crossflow being used (0.9 L/min, corresponding to a shear rate of approx. 2100 s^−1^), which was immediately corrected to 0.3 L/min (shear rate of 670 s^−1^), cell viability profiles are similar across all ATF runs performed (Figure 3B). Additionally, the different pore sizes applied (Table 1) did not result in any quantifiable effect on cell growth profiles (Figure 3A,B).

The profiles of AAV concentrations produced by ATF bioreactors are depicted in Figure 3C with values representing the sum of titers (VG/mL) obtained in each fraction (i.e., intracellular, extracellular, and permeate fractions) for a given time point. ATF1 and ATF3 show the same concentration profile, lower than ATF2. In addition, a plateau in concentration is reached after 50 h post-transfection irrespective of the ATF run, after which the concentration decreases. Importantly, the accumulated AAV titers per cell (VG/cell) in ATF2 and ATF3 are similar (approx. 7.6 × 10^4^ VG/cell), and almost 3-fold higher when compared to ATF1 (2.7 × 10^4^ VG/cell).

#### 3.2.2 Adeno-associated virus production using TFDF

**FIGURE 4:**
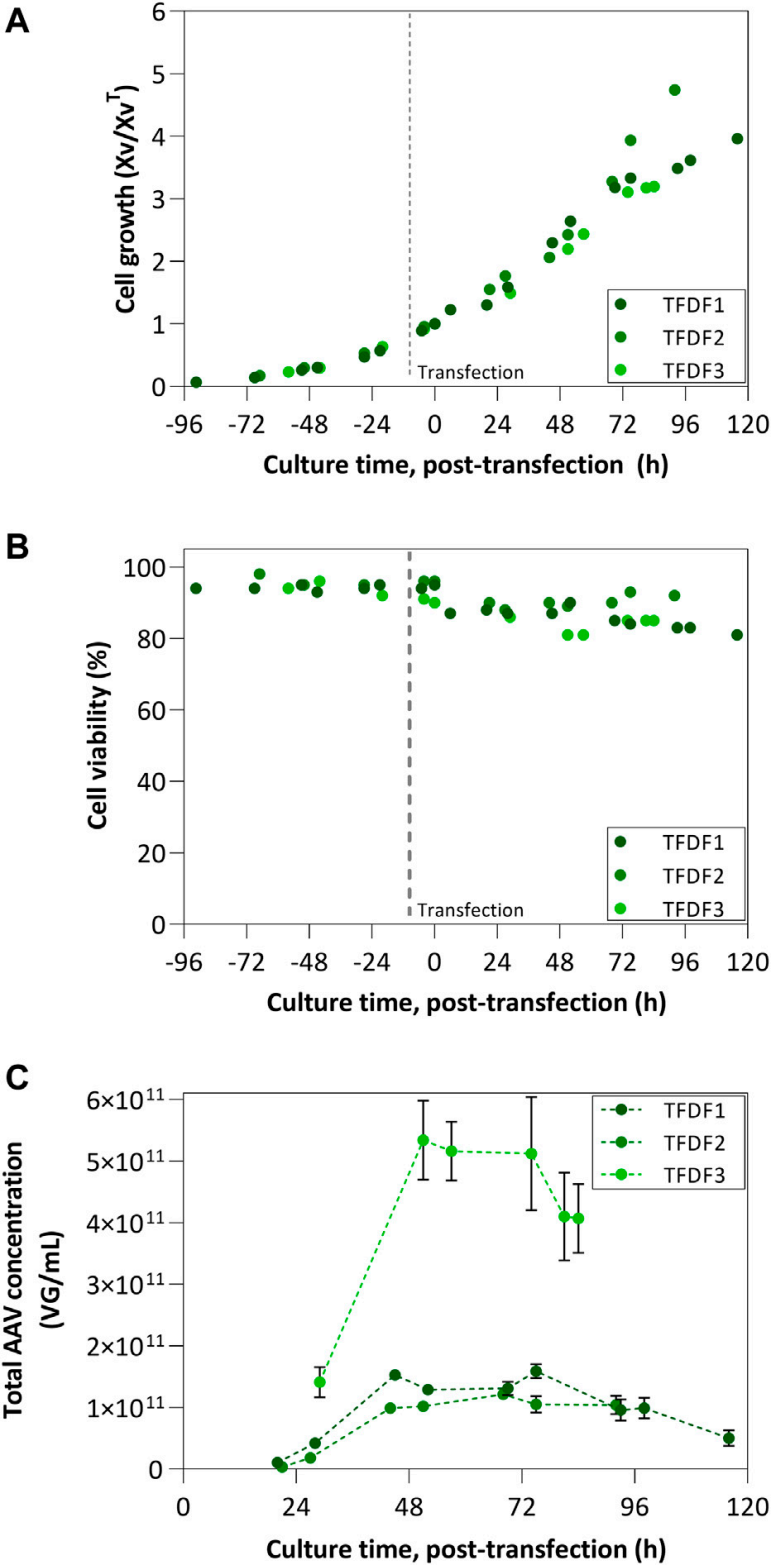
TFDF process results: **(A)** Cell growth profiles and **(B)** Cell viability profiles TFDF1 was transfected at 10 × 10^6^ cells/mL, while remaining runs transfected at 5 × 10^6^ cells/mL; Cell growth is represented by normalizing viable cell concentration by the VCC at the time of transfection; **(C)** Concentration of total AAV produced considering intracellular, extracellular and permeate fractions.

The normalized cell growth profiles were similar for all the TFDF runs (Figure 4A), with small changes at the end of the culture. The same observation is valid for the percentage of viable cells, remaining constant until the time of transfection, upon which a decrease is observed. Noticeably, although the shear rate used throughout TFDF1 run was the same as in ATF1 (approx. 2100 s^−1^), there was no apparent negative impact on cell viability (Figure 4B *vs.* Figure 3B).

Figure 4C reports the kinetics of AAV concentration in the bioreactor. AAV concentration reached a plateau in all three bioreactor runs at around 48 h post-transfection followed by a decrease in concentration towards the end of the culture. TFDF1 and TFDF2 show the same concentration profile, lower than TFDF3. Noteworthy, the accumulated AAV titers per cell (VG/cell) in TFDF3 are almost 4-fold higher than those obtained in TFDF1 and TFDF2 (8.2 × 10^4^ VG/cell *vs.* 1.6–2.2 × 10^4^ VG/cell).

### 3.3 Clarification and harvest of adeno-associated virus

#### 3.3.1 Adeno-associated virus produced in batch mode

The AAVs produced in bioreactors operated in batch mode were clarified post lysis using a two-stage filter scheme (see details in Experimental methods—Section 2) and the results are presented in Table 2. Both filters evaluated in the first stage achieved AAV recoveries above 90%, with TFDF having higher recovery yields and load (752 *vs.* 23 L/m^2^). The second filtration stage is characterized by having AAV recoveries in the range of 71–90%, with the Millistak filter presenting the highest recovery yield (90%) and load (approx. 290 L/m^2^). In summary, the combination of TFDF with Millistak resulted in a global AAV recovery of 90%. In addition, all filter stages reduced turbidity to levels below 30 NTU, with the trains using TFDF reaching a level below 10 NTU.

**TABLE 2:** Clarification of batch cultures. A two-stage filtration process was used; the filters in the first stage have the same pore size (5 µm); the second stage filters are depth filters.

Concentration at TOT (cells/mL)	Concentration at TOH (cells/mL)	First stage			Second stage			Clarification	Turbidity	
		Type	Load (L/m^2^)	VG yield (%)	Type	Load (L/m^2^)	VG yield (%)	Global VG yield (%)	After lysis	After filtration
2.6 × 10^6^	7.1 × 10^6^	ULTA GF (5.0 µm)	23	91	Sartopore 2 XLG (0.8/0.2)	30	82	72	480	26
5.6 × 10^6^	4.6 × 10^6^	TFDF (5.0 µm)	752	99	Sartopore 2 XLG (0.8/0.2)	34*	71	71	414	8
					Millistak HC X0SP	287	90	90	414	7

*Corresponds to a minimum load, performed with remaining material from TFDF (5.0 µm) + Millistak HC, X0SP, experiment; TOT, time of transfection; TOH, time of harvest; VG, viral genomes.

#### 3.3.2 Adeno-associated virus produced in perfusion mode

**FIGURE 5:**
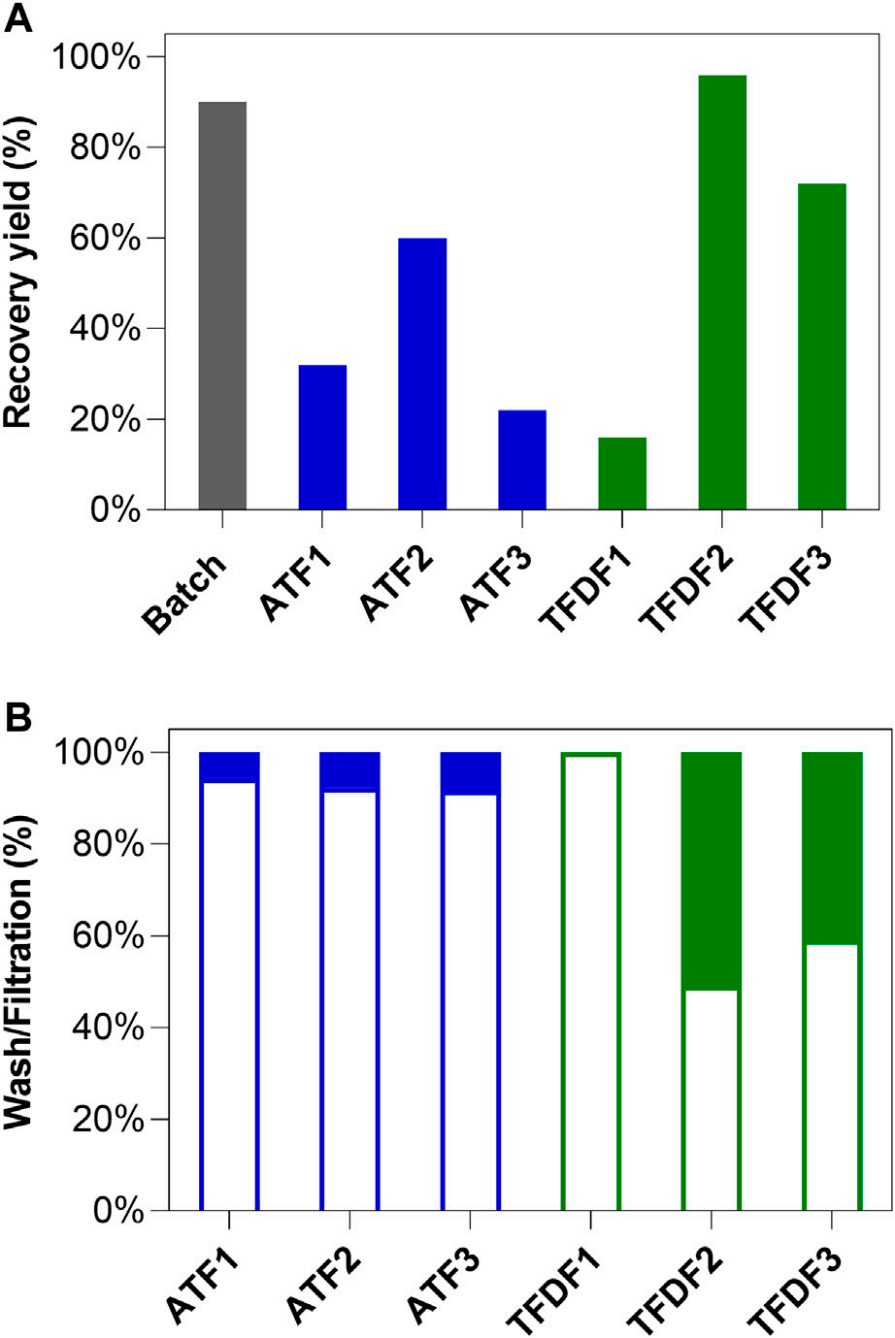
TFDF and ATF clarification process results: **(A)** Clarification yields from batch culture (grey bar), ATF (blue bars), and TFDF (green bars) runs; **(B)** Percentage of the recovered AAV during washing and filtration stages. The empty and full bars represent the recovery percentage of AAV in the wash and filtration stages respectively.

The AAV produced in bioreactors operated in perfusion mode were clarified as described in Experimental methods (Section 2) and results are presented in Table 3 and Table 4 and Figure 5.

**TABLE 3:** Clarification of perfusion cultures. The clarification process is composed of a wash step for recovering extra-cellular virus, followed by cell lysis, nuclease treatment and final filtration through the cell retention device.

Bioreactor	Load (L/m^2^)	Turbidity after cell lysis (NTU)	Turbidity after clarification (NTU)
**ATF1**	25	*NA*	*NA*
**ATF2**	17	1644	3
**ATF3**	16	2660	1
**TFDF1**	*NA*	4290	*NA*
**TFDF2**	581	860	668
TFDF3*	66 + 615	2100	1240

Turbidity after clarification was measured after ATF, or TFDF, filtration.

NA, not available (sample too turbid for measurement).

*Two filters were used in these experiments; each value corresponds to the throughput of each filter.

**TABLE 4:** Clarification process using TFDF combined with second stage filters.

Bioreactor	Concentration at TOH (cells/mL)	Second stage		Turbidity	Filters
		Load (L/m^2^)	VG yield (%)		
TFDF3	17.7 × 10^6^	33*	78	22	TFDF (5.0 µm) + Sartopore 2 XLG (0.8/0.2)
		64	92	7	TFDF (5.0 µm) + Millistak HC X0SP

*Corresponds to a minimum load; all available material was filtered with no pressure change.

The overall AAV recovery yield after clarification is reported in Figure 5A whereas the ratio between viruses harvested in the prior lysis wash and lysate clarification stages is reported in Figure 5B. ATF1 and ATF3 are the runs where lower AAV recoveries were observed (33% and 23%, respectively); ATF2 returned a recovery yield of 61% (Figure 5A). Noteworthy, all ATF runs are characterized by having more than 91% of the virus being recovered in the media exchange stage prior to lysis (Figure 5B). Regarding TFDF runs, TFDF1 reports the lowest recovery yields, with TFDF2 and TFDF3 enabling AAVs recovery yields of 97% and 73%, respectively (Figure 5A). Importantly, AAV in TFDF1 could only be harvested through the wash stage since, immediately after performing cell lysis, the AAV were no longer able to permeate through the membrane. In addition, results of TFDF3 were obtained with two TFDF primary filters since the substitution of the first was required after clogging was observed mid-run. TFDF2 and TFDF3 are characterized by a higher percentage of AAV being collected during the lysate clarification stage (51% and 41%, respectively).

Table 3 summarizes the calculated filter load for each ATF and TFDF run, and the turbidity values after cell lysis and clarification. ATF runs have a calculated load in the range of 16–25 L/m^2^, lower than those obtained in the TFDF experiments (66–615 L/m^2^). Regarding turbidity, ATF runs return the lowest turbidity values after cell lysis and clarification, with values ranging from 1–3 NTU. Knowing that the pore size of TFDF filters (2–5 µm) is substantially different from that of ATF filters (0.2 and 0.5 µm), a follow-up experiment was run in which secondary filtration was evaluated after TFDF using filters with a lower pore cutoff (similar to batch clarification scheme) for a fair comparison between TFDF and ATF. The results are summarized in Table 4 and demonstrate that both secondary filters enabled a turbidity reduction to levels close to those achieved in ATF runs (7–22 NTU), with AAV recoveries in the range of 78–92%.

## 4 Discussion

The main goal of this work was to evaluate ATF and TFDF devices for implementing perfusion cell cultures and to promote an integrated harvest and clarification strategy for AAV production.

The initial experiments in 2 L stirred-tank bioreactors operated in batch mode demonstrate the impact of increased culturing times, medium saturation, and higher cell densities on AAV production. These profiles are comparable to other HEK 293 cultures operated batchwise (Koh et al., 2009; Gálvez et al., 2012; Liste-Calleja et al., 2013). The potential limitation of nutrients and/or the accumulation of toxic by-products had a negative effect on specific productivities, this being more pronounced at higher VCC^TOT^ with a 5-fold lower specific production in BR5 (VCC^TOT^ = 5.57 × 10^6^ cells/mL) when compared to BR2 (VCC^TOT^ = 2.61 × 10^6^ cells/mL). The advantages of culture medium exchange towards the implementation of perfusion strategies were demonstrated by the results obtained in the small-scale experiments study. For the transfections performed in set A (batch mode) and set B (perfusion-mocking mode), the correlation between medium exchange at transfection and higher AAV production is clear. Although the AAV titers obtained in batch cultures are within reported ranges for this production system (extending from 0.1–3.58 × 10^4^ VG/cell) (Blessing et al., 2019; Guan et al., 2022), these were considerably lower (up to 3−fold) than the ones obtained with perfusion-mocking. This set of experiments shows the potential advantages of implementing perfusion methodologies and corroborates other reported studies on the subject (Vázquez-Ramírez et al., 2018; Fernandes et al., 2021; Escandell et al., 2022). Nevertheless, some optimization is still possible through 1) optimizing medium exchange rates to sustain VCC^TOT^ of 10 × 10^6^ cells/mL and balance the nutrient consumption associated with doubling the cell concentration after transfection (Fernandes et al., 2021), 2) exploring different plasmid ratios, proportions of total plasmid DNA and transfection reagent per cell (Chahal et al., 2014; Meade et al., 2021; Wosnitzka et al., 2021.), 3) engineering cell lines or use of different expression systems (Pais et al., 2020; Coronel et al., 2021) and/or 4) evaluating different bioreaction platforms, shown for other vectors to impact on particle quantity and quality regarding infectivity (Sousa et al., 2015; Blessing et al., 2019).

The characterization and comparison of perfusion modalities ATF and TFF have been described in the literature (Karst et al., 2016; Wang et al., 2017). Reports about the impact of shear stress on cell growth suggest that TFF devices are more prone to induce cell death and, considering the unidirectional flow of TFF in comparison to the bidirectional of ATF, faster to clog (Karst et al., 2016). This can be alleviated by, for example, using a centrifugal pump instead of a peristaltic one (Wang et al., 2017), similar to what is used in the TFDF system. In our study, the crossflow initially used in ATF1 impacted negatively on cell viability, but after reducing crossflow to one-third of the initial setting, the percentage of viable cells recovered to the initial values. This effect was not visible in any of the other ATF runs as well as the TFDF runs, despite some having similar shear rates (i.e., TFDF1 and TFDF2). Regarding AAV production, and after fine-tuning the crossflow using engineering correlations (Zhan et al., 2020), ATF and TFDF devices led to similar outcomes, i.e., changing VCC^TOT^ to half of the initial value (from 10 to 5 × 10^6^ cells/mL) concomitant with lower crossflow improves AAV titers per cell up to 3–4 fold. The particularity of TFDF, with the larger pore size (2.0–5.0 µm), could overcome the limitations reported for standard hollow fiber TFF by enabling the continuous permeation of larger impurities and harvest of products that could impair culture viability at higher cell densities (Wang et al., 2017). Given the results obtained, and the literature reports for the simpler TFF hollow-fibers, the TFDF device could be a more robust alternative for AAV production.

ATF and TFDF systems were also evaluated as the primary filtration stage in integrated clarification. For the continuous harvest of extracellular viruses, produced and permeated during perfusion cultures or during the washing step, ATF and TFDF enabled recoveries of 61% and 100% respectively, demonstrating a better performance with TFDF. For the clarification of lysates, the differences in ATF and TFDF characteristics are further evidenced, especially in turbidity reduction. Indeed, ATF experiments enable lower final turbidity values (<10 NTU), but this comes at the cost of lower AAV recovery yields obtained under the filtration stage of the defined clarification procedure. Studies reported in the literature with different pore sizes, similar to ATF, corroborate these results (Raghavan et al., 2019). Importantly, despite the sub-micron pore size of these membranes, the filtration performance was independent of cell concentration at the time of harvest. The same was not verified for all TFDF runs, with TFDF1 filter becoming severely fouled after cell lysis as a result of the high cell concentration at the time of harvest (approx. 50 × 10^6^ cells/mL compared to the 23 × 10^6^ cells/mL of ATF1) and/or the high concentration of impurities (Hadpe et al., 2017; Wang et al., 2017). Reducing cell concentration at the time of transfection (TFDF2 and TFDF3) led to a decrease in cell concentration and impurities at the time of harvest, thus impacting positively on the clarification step (i.e., no filter clogging/fouling).

## 5 Conclusion

The work herein reported demonstrates the potential of using ATF and TFDF for high-yield production of AAV8 using mammalian HEK293T cells and transient transfection in STB. Shear rate is a key factor in ATF or TFDF process implementation, impacting negatively on cell growth and virus expression kinetics, and thus require fine-tuning to maximize AAV production. Matching the apparent shear rate found in ATF with that of TFDF, AAV specific titers could be improved by up to almost 4-fold in TFDF and surpass ATF and perfusion-mocking experiments carried in shake flasks. In clarification, both ATF and TFDF enable a continuous harvest of the extracellular viruses during the production and washing phases but with a better performance measured for TFDF. Filtration of cell lysates could only be achieved with TFDF. In summary, the AAV titers and clarification yields obtained with TFDF demonstrated the capabilities of this technique for continuous integrated production, harvest, and clarification of AAV and potentiate further developments in high cell density and intensified processes.

## Data Availability

The original contributions presented in the study are included in the article/supplementary material, further inquiries can be directed to the corresponding author.
